# Supplementary material for: Label-free hyperspectral imaging and deep-learning prediction of retinal amyloid β-protein and phosphorylated tau
Source: PNAS Nexus. 2022 Aug 19;1(4):pgac164. doi: 10.1093/pnasnexus/pgac164 (PMC9491695; doi:10.1093/pnasnexus/pgac164)
Supplement: pgac164_Supplemental_File [file pgac164_supplemental_file.docx]

Supporting Information

**Label-Free Hyperspectral Imaging and Deep-Learning Prediction of Retinal Amyloid β-Protein and Phosphorylated Tau**

*Xiaoxi Du^1^, Yosef Koronyo^2^, Nazanin Mirzaei^2^,* *Chengshuai Yang^1^, Dieu-Trang Fuchs^2^, Keith L. Black^2^, Maya Koronyo-Hamaoui*^2,3^, Liang Gao*^1^*

^1^Department of Bioengineering, University of California Los Angeles, Los Angeles, CA 90095, USA

^2^Department of Neurosurgery, Maxine Dunitz Neurosurgical Research Institute, Cedars-Sinai Medical Center, Los Angeles, CA, 90048, USA

^3^Department of Biomedical Sciences, Division of Applied Cell Biology and Physiology, Cedars-Sinai Medical Center, Los Angeles, CA, 90048, USA

*Equal contributors & Corresponding authors: [gaol@ucla.edu](mailto:gaol@ucla.edu); maya.koronyo@csmc.edu

**Experimental Section S1: Hyperspectral Imaging System Configuration**


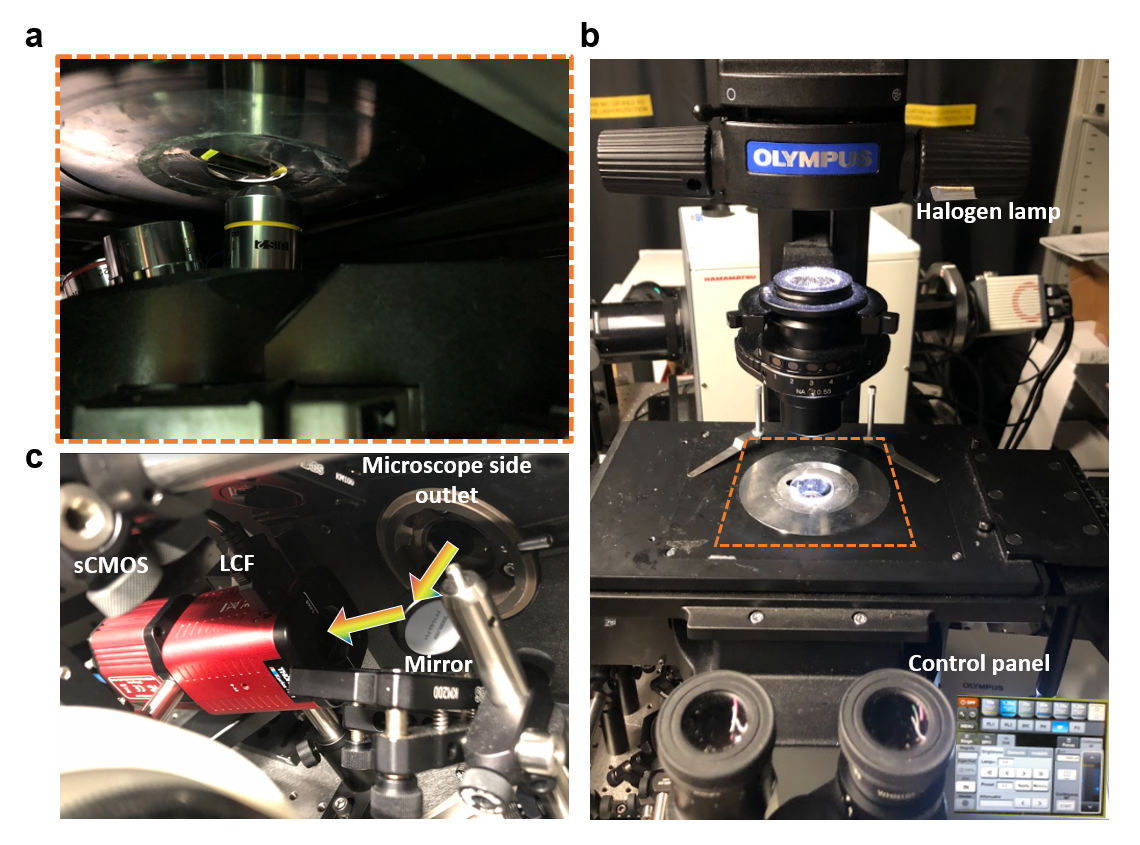


**Figure S1. Hyperspectral microscope. a.** Transmission imaging mode by a 10x objective. The objective is placed under the sample stage circled in orange dashed area in b**. b.** The hyperspectral microscope with a halogen lamp illumination above the sample stage, the illumination and stage coordinates parameters and corresponding control panel. **c.** Side view of the outside microscope. The light collected by the objective is relayed to the outside of the microscope and reflected by a mirror into liquid crystal filter (LCF) head. The filtered light is transmitted through an optical tube to a sCMOS camera.

**Experimental Section S2: Aβ_42_ and pS396-Tau Spectral Signatures in Different Retinal Layers**


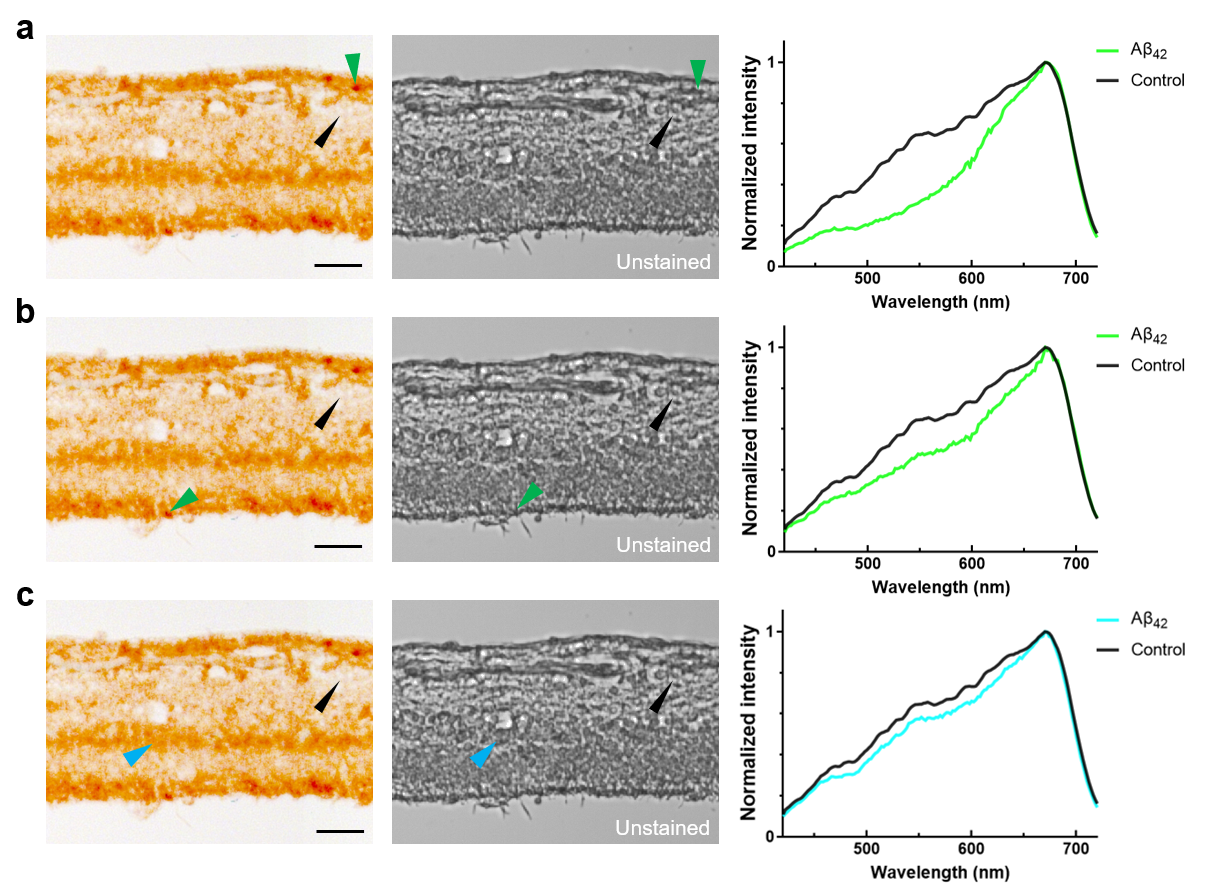


**Figure S2. Hyperspectral imaging of various** **retinal Aβ_42_ deposits and locations. a.** 12F4^+^-Aβ_42_ deposits at ganglion cell layer (GCL). **b.** Aβ_42_ deposits in photoreceptors. **c.** Diffusive Aβ_42_ deposits in retinal OPL. From left to right, DAB-labeled Aβ_42_ retina cross-sections, unstained hyperspectral intensity images, spectra at arrow-pointed locations. Black arrows: Control regions. Green and Blue arrows: targeting Aβ_42_ regions. Scale bar, 50 µm.


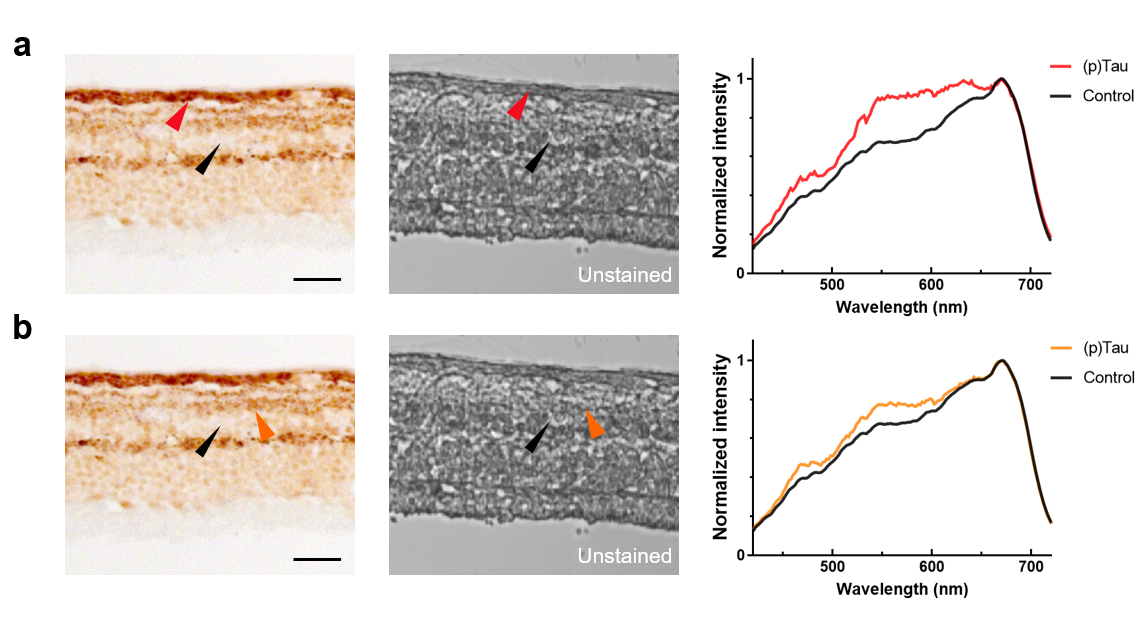


**Figure S3. Hyperspectral imaging of various pS396-Tau deposits locations. a.** pS396-Tau deposits at innermost retinal layers. **b.** Diffusive pS396-Tau deposits in inner plexiform layer (IPL), forming three distinct bands. Diffused pS396-Tau spectrum has the same characteristic with aggregated pS396-Tau but has reduced magnitude. From left to right, DAB labeled pS396-Tau retinal cross-sections, unstained hyperspectral intensity images, spectra at arrow-pointed locations. Black arrows: Control regions. Red and orange arrows: targeting pS396-Tau regions. Scale bar, 50 µm.


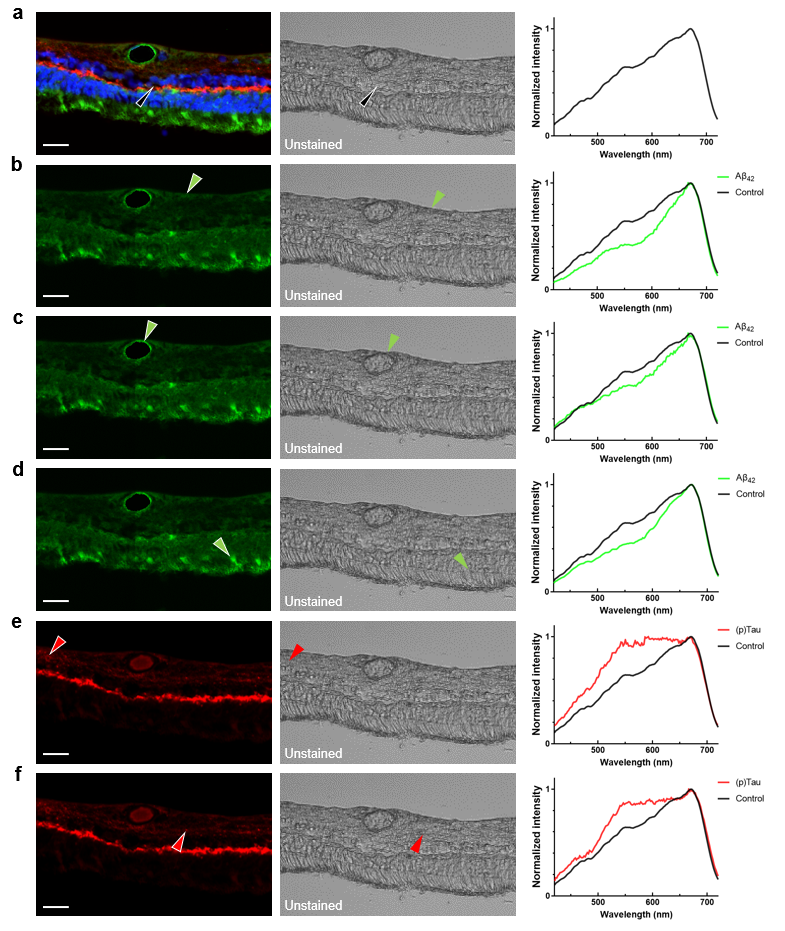


**Figure S4. Hyperspectral imaging of various retinal Aβ_42_ and pS396-Tau deposits and locations. a.** Merged immunofluorescence-stained retinal cross-section image and corresponding HSI retinal image. A normal tissue region was selected as control for other 12F4^+^-Aβ_42_ and pS396-Tau deposits analysis. **b.** Aβ_42_ immunofluorescence-stained channel, with arrow pointed at Aβ_42_ deposits in nerve fiber layer (NFL). **c.** Aβ_42_ immunofluorescence-stained channel, with arrow pointed at Aβ_42_ deposits in the vascular wall. **d.** Aβ_42_ immunofluorescence-stained channel, with arrow pointed at Aβ_42_ deposits in photoreceptors. **e.** pS396-Tau immunofluorescence-stained channel, with arrow pointed at pS396-Tau deposits in neurofibrillary tangle (NFT)-like structures in GCL. **f.** pS396-Tau immunofluorescence-stained channel, with arrow pointed at aggregated pS396-Tau deposits in the three distinct bands of IPL. From left to right, immunofluorescence-stained retinal cross-sections, unstained hyperspectral intensity images, spectra at arrow-pointed locations. Black arrows: Control regions. Green and red arrows: targeting Aβ_42_ and pS396-Tau regions, respectively. Scale bar, 50 µm.


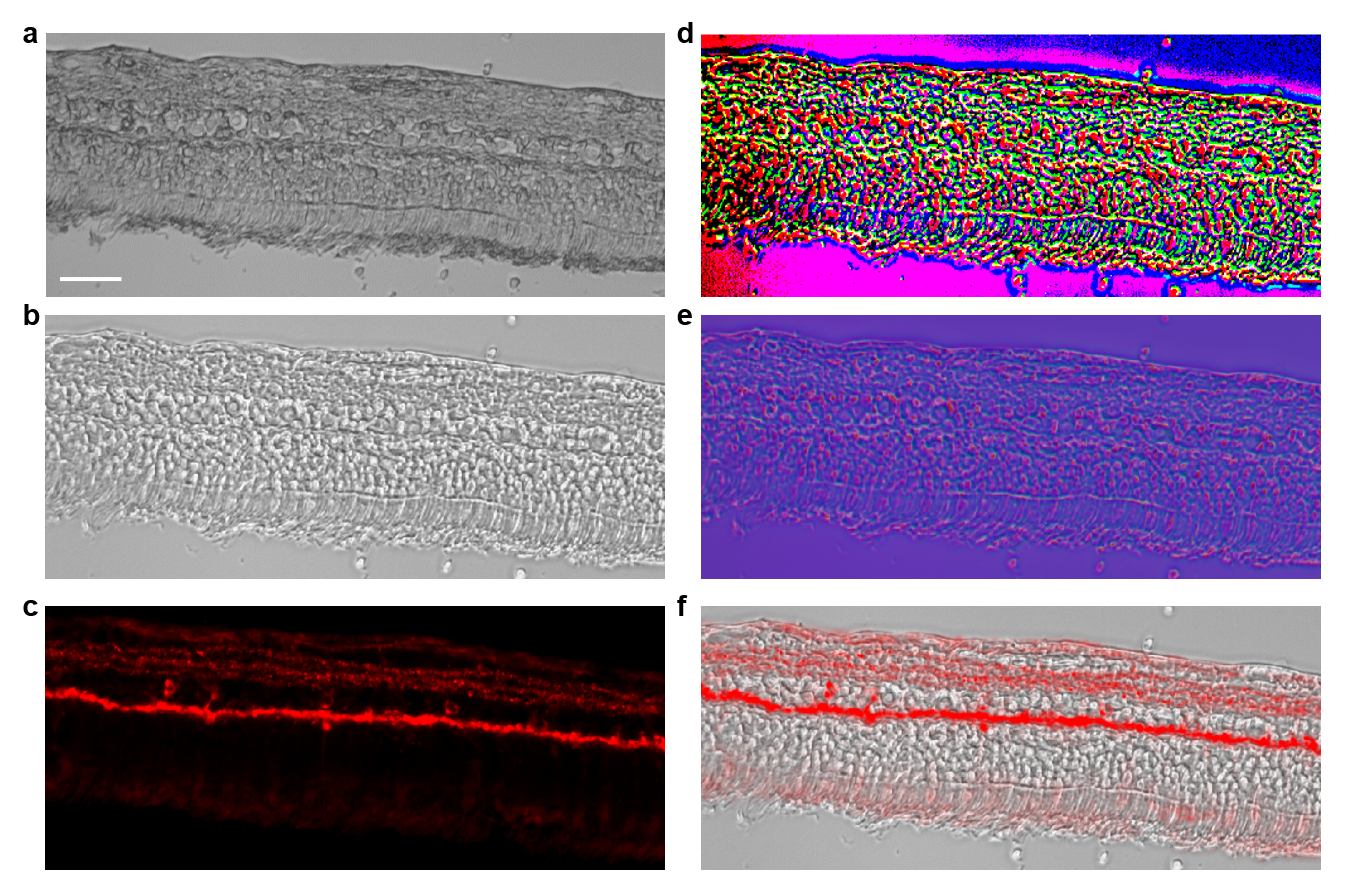


**Figure S5. Hyperspectral image processing pipeline. a.** Raw hyperspectral image. **b.** Normalized hyperspectral image**. c.** Corresponding ground truth image with pS396-Tau labeling. **d.** Principal component analysis (PCA) processed image through normalized image b. **e.** PCA-HSI retinal image after shifting and stretching the first three principal components. **f.** Hyperspectral image with pS396-Tau labeling by image registration (b and c). Gray channel: normalized hyperspectral image. red channel: pS396-Tau label. Scale bar, 50 µm.

**Experimental Section S3: Aβ42 and pS396-Tau Spectral Consistency Measured over An Extended Time Period**

**
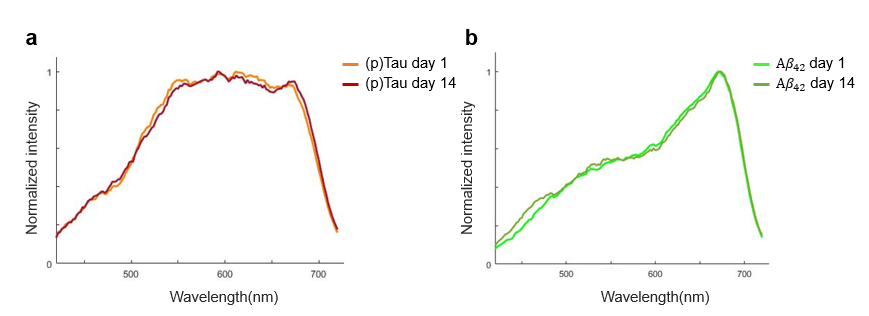
**

**Figure S6. Spectra of (a) Aβ_42_ and (b) pS396-Tau of the same sample measured on day 1 and day 14.**

**Experimental Section S4: Additional Transformed Retinal Images**


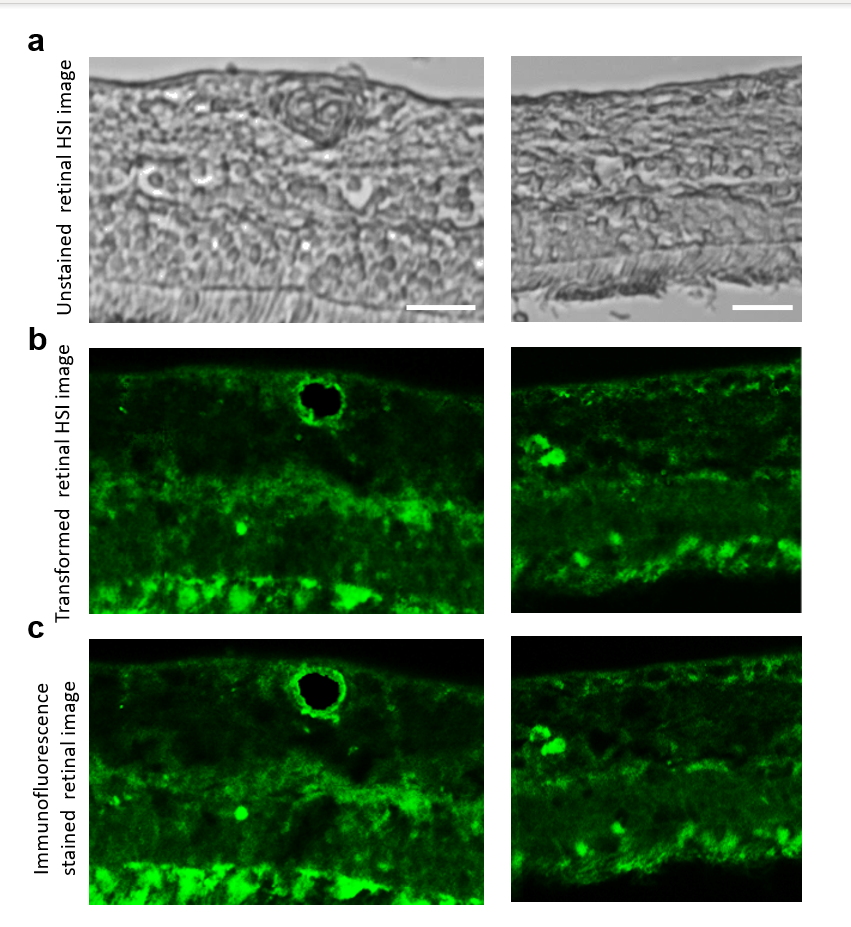


**Figure S7. Histopathology prediction of hyperspectral images with the** **immunofluorescence-****Aβ_42_ model. a.** Unstained hyperspectral intensity images of retinal cross-sections. **b.** Transformed immunofluorescence-Aβ_42_ labeled retinal image by stitching the output patches from the network. **c.** Real immunofluorescence stained Aβ_42_ retinal images. Left and right columns: two different field-of -views (FOV). Scale bar, 50 µm.


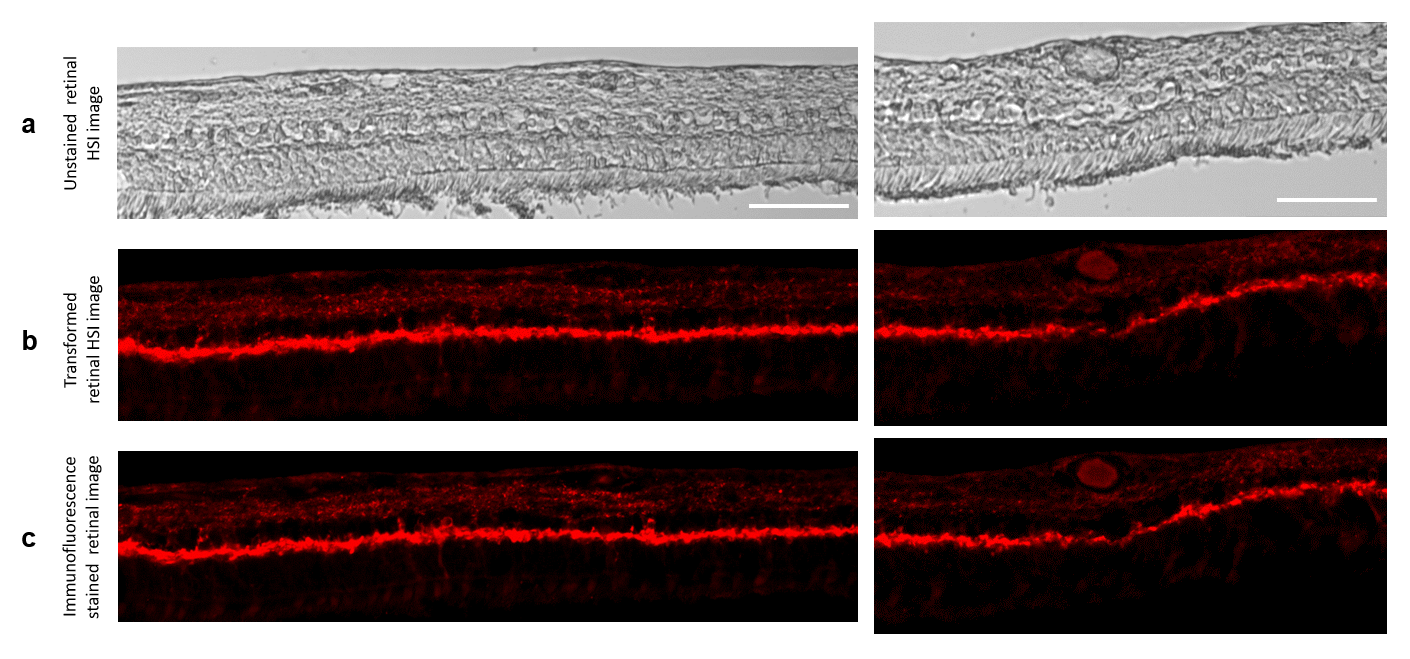


**Figure S8. Histopathology prediction of hyperspectral images with the immunofluorescence-pS396-Tau model. a.** Unstained hyperspectral intensity images of retinal cross-sections. **b.** Transformed immunofluorescence-pS396-Tau labeled retinal image by stitching the output patches from the network. **c.** Image of real immunofluorescence stained retinal pS396-Tau. Left and right columns: two different FOVs. Scale bar, 100 µm.


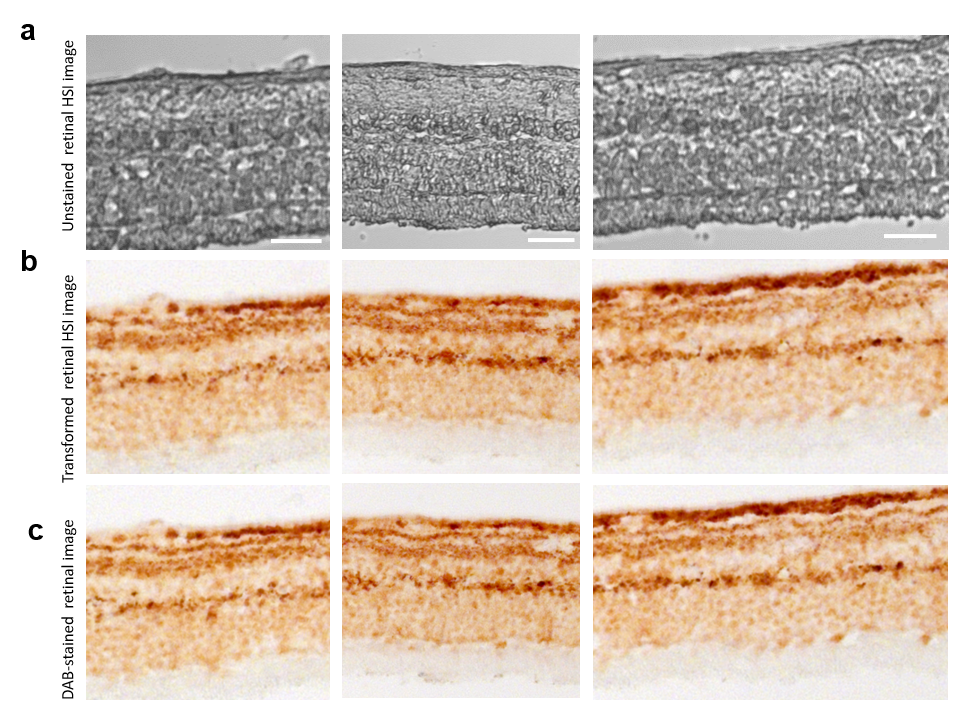


**Figure S9. Histopathology prediction of hyperspectral images with the DAB-pS396-Tau model. a.** Unstained hyperspectral intensity images of retinal cross-sections. **b.** Transformed DAB- pS396-Tau labeled retinal image by stitching the output patches from the network. **c.** Real DAB-stained pS396-Tau retinal images. Left, middle, and right columns: three different FOVs. Scale bar, 50 µm.


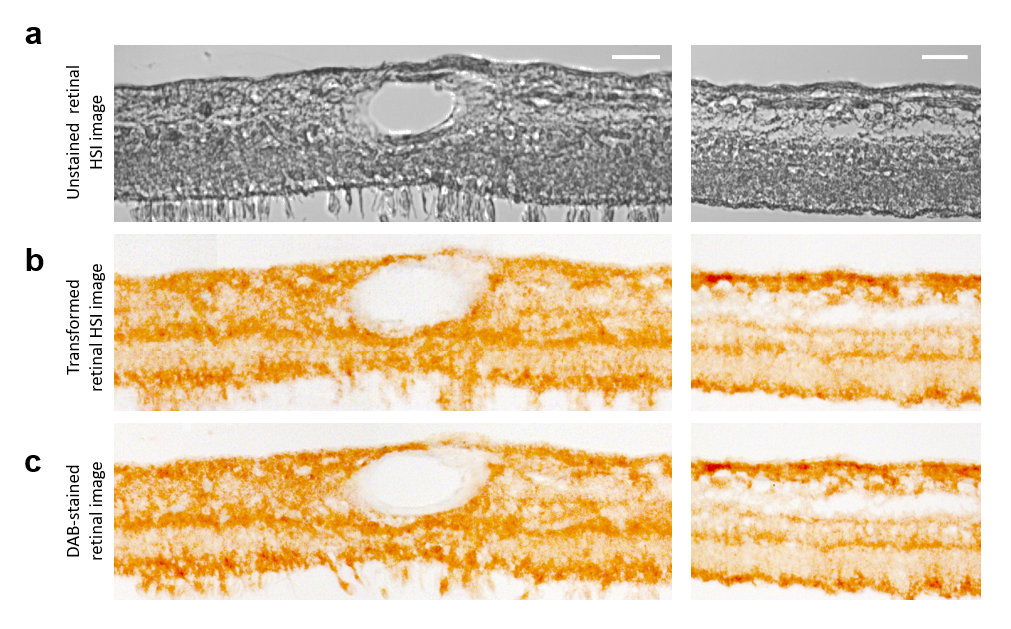


**Figure S10. Histopathology prediction of hyperspectral images with the DAB-Aβ_42_ model. a.** Unstained hyperspectral intensity images of retinal cross-sections. **b.** Transformed DAB-Aβ_42_ labeled retinal image by stitching the output patches from the network. **c.** Real DAB-stained Aβ_42_ retinal images. Left, middle, and right columns: three different FOVs. Scale bar, 50 µm.

**Table S1. Evaluation statistics by structural similarity (SSIM) index and peak signal-to-noise ratio (PSNR).**

| Model | SSIM | | PSNR | |
| --- | --- | --- | --- | --- |
|  | Average | Standard deviation | Average | Standard deviation |
| DAB-pS396-Tau | 0.8714 | 0.0122 | 23.3136 | 1.2700 |
| DAB-Aβ_42_ | 0.8203 | 0.0136 | 21.1323 | 1.2910 |
| Fluo-pS396-Tau | 0.8326 | 0.0171 | 32.9626 | 1.0297 |
| Fluo-Aβ_42_ | 0.8128 | 0.0219 | 26.3196 | 1.6265 |

**Table S2. The number of 256×256 pixels patches used in training for the four transformation models.**

| Transformation model | DAB-pS396-Tau | DAB-Aβ_42_ | Fluo-pS396-Tau | Fluo-Aβ_42_ |
| --- | --- | --- | --- | --- |
| # of training patches | 16528 | 29630 | 13501 | 13706 |
| # of validation patches | 360 | 650 | 300 | 300 |
| # of test patches | 398 | 713 | 324 | 330 |

**Experimental Section S5: Retina and Brain Neuropathological Assessments**

Amyloid plaques and tangles in the brain were evaluated using anti–β-amyloid mAb clone 4G8, Thioflavin-S (ThioS), and Gallyas silver stain in formalin-fixed, paraffin-embedded tissues. Two neuropathologists provided scores based on independent observations of β-amyloid, NFT burden, and/or neuropil threads (0 = none; 1 = sparse 0–5; 3 = moderate 6–20; 5 = abundant/frequent 21–30 or greater; or N/A= not applicable), and an average of two readings was assigned to each individual patient. A final diagnosis included AD neuropathological change. The Aβ plaque (A) score was modified from that proposed by Tal et al. (A0 = no Aβ or amyloid plaques; A1 = Thal phase 1 or 2; A2 = Thal phase 3; or A3 = Thal phase 4 or 5).(1) The NFT (B) stage was modified from that offered by Braak for silver-based histochemistry or p-tau IHC (B0 = no NFTs; B1 = Braak stage I or II; B2 = Braak stage III or IV; or B3 = Braak stage V or VI)(2) and the neuritic plaque (C) score was modified from CERAD (C0 = no neuritic plaques; C1 = CERAD score sparse; C2 = CERAD score moderate; or C3 = CERAD score frequent).(3)

**Table S3. The** **neuropathologically confirmed patients used for validation of immunofluorescence and DAB imaging.**

| Neuropathological diagnosis | Patient # | Age (year) | Gender | Race |
| --- | --- | --- | --- | --- |
| AD | #1 | 90 | Female | White |
| AD | #2 | 92 | Female | Hispanic |
| MCI | #1 | 93 | Male | White |
| MCI | #2 | 87 | Female | White |
| MCI | #3 | 88 | Male | White |
| CN | #1 | 72 | Male | White |
| CN | #2 | 84 | Male | White |
| CN | #3 | 95 | Female | White |
| CN | #4 | 77 | Male | White |
| CN | #5 | 95 | Female | White |

**Table S4. The** **neuropathologically confirmed AD patients used for spectral signature analysis and transformation network training. Retina cross-sections stained for Amyloid beta 42 (Ab 12F4) and pS396-Tau (Ab PS396). Postmortem interval (PMI) values of tissue collection were also recorded.**

| Donor information | Patient #1 | Patient #4 | Patient #5 |
| --- | --- | --- | --- |
| Age (year) | 90 | 81 | 85 |
| Gender | Female | Female | Female |
| Race | White | Hispanic | White |
| Premortem diagnosis | AD | AD | Dementia |
| MMSE score | 9 | None | None |
| CDR score | 2 | 3 | 3 |
| PMI (hour) | 9 | 7.5 | 8.5 |
| Final diagnosis | AD | AD | AD |
| Braak stage | V | VI | V |
| ADNC: Aβ plaque (A) score, NFT (B) stage and neuritic plaque (C) score | A2, B3, C3 | A3, B3, C3 | A3, B3, C3 |
| HSI imaged slide # | #05 | #12 | #04 |
| Fluorescent stained slide # | 12F4 & PS396: #06 | 12F4 & PS396: #13, #12 | 12F4 & PS396: #03 |
| DAB stained slide # | 12F4: #05, #08  PS396: #04 | 12F4: #11  PS396: #14 | 12F4: #05  PS396: #04, #01 |
| Transformation model | DAB-Aβ_42_ | Fluo-Aβ_42_  Fluo-pS396-Tau | DAB-pS396-Tau |

**References:**

1. D. R. Thal, U. Rüb, M. Orantes, H. Braak, Phases of A beta-deposition in the human brain and its relevance for the development of AD. *Neurology* **58**, 1791–1800 (2002).

2. H. Braak, I. Alafuzoff, T. Arzberger, H. Kretzschmar, K. Tredici, Staging of Alzheimer disease-associated neurofibrillary pathology using paraffin sections and immunocytochemistry. *Acta Neuropathol* **112**, 389–404 (2006).

3. S. S. Mirra, *et al.*, The Consortium to Establish a Registry for Alzheimer’s Disease (CERAD). Part II. Standardization of the neuropathologic assessment of Alzheimer’s disease. *Neurology* **41**, 479–486 (1991).
